# Rate of Freeze Impacts the Survival and Immune Responses Post Cryoablation of Melanoma

**DOI:** 10.3389/fimmu.2021.695150

**Published:** 2021-06-03

**Authors:** Chakradhar Yakkala, Julien Dagher, Christine Sempoux, Cheryl Lai-Lai Chiang, Alban Denys, Lana E. Kandalaft, Bhanu Koppolu, Rafael Duran

**Affiliations:** ^1^ Department of Radiology and Interventional Radiology, Lausanne University Hospital and University of Lausanne, Lausanne, Switzerland; ^2^ Institute of Pathology, Lausanne University Hospital and University of Lausanne, Lausanne, Switzerland; ^3^ Department of Oncology, Lausanne University Hospital, Ludwig Institute for Cancer Research, University of Lausanne, Lausanne, Switzerland; ^4^ Center of Experimental Therapeutics, Department of Oncology, Lausanne University Hospital, Lausanne, Switzerland; ^5^ Interventional Oncology and Immuno-oncology, BTG/Boston Scientific, Natick, MA, United States

**Keywords:** cryoablation, melanoma, immunotherapy, immune response, tumors

## Abstract

The emergence of ablative therapies has revolutionized the treatment of inoperable solid tumors. Cryoablation stands out for its uniqueness of operation based on hypothermia, and for its ability to unleash the native tumor antigens, resulting in the generation of anti-tumor immune responses. It is not clearly understood how alterations in the rate of freeze impact the immune response outcomes. In this study, we tested fast freeze and slow freeze rates for their locoregional effectiveness and their ability to elicit immune responses in a B16F10 mouse model of melanoma. Tumor bearing mice treated with fast freeze protocol survived better than the ones treated with slow freeze protocol. Fast freeze resulted in a higher magnitude of CD4^+^ and CD8^+^ T-cell responses, and a significantly extended survival post re-challenge. Thus, fast freeze rate should be applied in any future studies employing cryoablation as an *in vivo* vaccination tool in conjunction with targeted immunotherapies.

## Introduction

Cryoablation is increasingly applied in the clinics for the local management of tumors, including malignancies of kidney, lung, soft tissues and bone cancers. Cryoablation proved its ability to effectively eradicate small tumors inoperable by surgery. In contrast to surgery which totally removes tumor tissues, *in situ* ablation of tumors also releases tumor antigens into systemic circulation, eliciting immune responses against tumor-specific and tumor-associated antigens (1).

Cryoablation results in a higher magnitude of post-ablative immunogenic responses as compared to radiofrequency ablation or laser induced thermotherapy, which could be partly due to the preservation of tumor antigenic structures that are otherwise denatured in hyperthermic ablation procedures (2). The immune responses post-ablation were suggested as the underlying mechanism for the spontaneous regression of metastases occasionally observed post cryoablation of primary tumors (3–5). This immunogenic potential along with its minimal invasiveness, repeatability, and relatively painless properties makes cryoablation an attractive option to be combined with targeted immunotherapies in patients with metastatic disease, in order to achieve improved systemic anti-tumor immune responses. Indeed, co-application of cryoablation with TLR agonists, adoptive immune cell therapies and checkpoint inhibitors have been shown to be beneficial in various tumor mouse models and patients with cancer (6–10).

Rate of freeze is a crucial factor that dictates the effectiveness of cryoablation (11). Fast and slow freezing rates have long been suggested to vary in their biophysical processes of inducing cell injury (12). Fast freeze rate is employed for the locoregional treatment of solid tumors in the clinic. Nevertheless, the effect of freeze rate on the mode of tissue death (i.e. apoptosis *vs* necrosis) and its impact on the subsequent immune responses, remains largely elusive to date. Our study aims to address these aspects, which will be pivotal in the context of employing cryoablation as an *in vivo* vaccination tool for cryo-immunotherapies. To increase the translational relevance of our study, we used a clinical cryoablation system, and compared fast and slow freeze rates in their locoregional effectiveness on tumor growth and viability, and their ability to generate immune responses in a B16F10 mouse model of melanoma. We performed an extensive immune cell phenotyping by flow cytometry of tumors, tumor draining and non-draining lymph nodes. Our results demonstrate significant changes in the overall frequencies of CD4^+^ and CD8^+^ T-cell subsets in the lymph nodes following cryoablation, and are more pronounced in the fast freeze mode of ablation. Re-challenge experiments resulted in an extended survival of mice treated with fast freeze protocol (in comparison to non-treated controls), indicative of tumor-specific immune responses elicited post cryoablation. Taken together, our data suggests an advantage of fast freeze over slow freeze rate in the locoregional tumor control and the generation of immune responses.

## Materials and Methods

### Tumor Cell Line

B16F10 melanoma cells (Ludwig Institute for Cancer Research (LICR), Lausanne) were cultured in Dulbecco’s modified Eagle’s medium (DMEM) (31966-021, gibco), supplemented with 10% fetal bovine serum (FBS) (10270, gibco) and 1% penicillin-streptomycin (15140-122, gibco), maintained at 5% CO_2_ and 37°C.

### Animal Model

C57BL/6JOlaHsd female mice were purchased from Envigo (Gannat, France). Animals were maintained in specific pathogen-free conditions. Experiments were performed as per the institutional guidelines, approved by Service de la Consommation et des Affaires Vétérinaires, Vaud, Switzerland. Animals were allowed to acclimatize for at least one week prior the start of experiments. Mice were given food and water *ad libitum*. 8-10 week old mice were shaved on the flank, and transplanted subcutaneously with 5 x 10^6^ B16F10 cells re-suspended in 100µl phosphate buffer saline (PBS). The day of transplant is considered as day 1 time point. For re-challenge experiments post cryoablation, mice received 5 x 10^6^ B16F10 cells in a similar manner on the contralateral flank. Tumor growth was measured by electronic vernier calipers, and the volume was calculated by the formula: (width^2^ x length)/2.

### Experimental Design

Mice with tumors were randomized into 3 groups: (a) non-treated group, (b) slow-freeze group, and (c) fast-freeze group. Tumor growth was measured in all animals on the day of cryoablation (groups were well balanced with no statistically significant differences in tumor size at baseline), followed by every 2 days post ablation, alongside the non-treated controls. Mice that developed tumors ≥1000mm^3^ volume or showed severe impairment were sacrificed as per the local regulations. Surviving mice that were free of tumors were subjected to re-challenge experiments 2 months post primary B16F10 transplant. For analyses by flow cytometry and immunohistochemistry, animals were randomly sacrificed at day 3-4 or 7-8 post-cryoablation.

For the analysis of plasma cytokines, blood was extracted from the tail vein on 2 days prior cryoablation, followed by 3 days or 8 days post cryoablation. Blood was collected in anticoagulant coated tubes, centrifuged, and the supernatant plasma was collected and frozen at -80^0^C for further cytokine analyses. Tumor necrosis factor-α (TNF-α), interleukin (IL)-1α, IL-1β, IL-12p70, IL-10, IL-6, IL-17α, IFN-β, IFN-γ, IL-23, IL-27, monocyte chemoattractant protein-1, and granulocyte monocyte colony stimulating factor were measured by Legendplex kit as per the manufacturer’s instructions (740446, Biolegend).

### Cryoablation

Tumors were cryoablated at day 7-9 post B16F10 cell transplant. Mice were anesthetized by 2-2.5% isoflurane inhalation. The skin above the tumor and its surrounding area was shaven, disinfected with 70% ethanol. An incision was created on the skin adjacent to the tumor in order to expose the tumor for cryoablation. To increase the translational value of gathered data, we utilized a cryomachine used in the clinic (Visual ICE System BTG, Boston Scientific). The cryoprobe was inserted into the tumor followed by a freeze-thaw process. Freeze step involved the release of high-pressure Argon gas through the needle into the tumor until the tumor was completely frozen (macroscopic ice ball encapsulates the tumor). Thaw step involved allowing the tumor to passively defrost and return to its previous state. All the cryoablations involved two freeze-thaw cycles. Fast freeze indicates 100% freeze intensity (uninterrupted Argon flow) as opposed to slow freeze that represents 5% freeze intensity (i.e., Argon is pumped for 0.5 seconds in every 10 second time period). Surgical wounds were closed with non-absorbable sutures. Paracetamol was given to mice in drinking water (2mg/ml) as an analgesic for a period of one week post-cryoablation.

### Flow Cytometry of Tumors and Lymph Nodes

Tumor tissues (ablated and non-ablated) were digested by mouse tumor dissociation kit (130-096-730, Miltenyi Biotec) as per the manufacturer’s instructions in a gentleMACS™ octodissociator (130-096-427, Miltenyi Biotec), under 37C_m_TDK_2 program. Tumor lysates were subjected to red blood cell (RBC) lysis by 1X RBC lysis buffer (00-4333-57, Thermo Fisher Scientific). As a final step, tumor cells were re-suspended in PBS and filtered through 70µm MACS smart strainers (130-110-916, Miltenyi Biotec) to obtain single cell suspensions. Tumor draining and non-draining (contralateral) inguinal lymph nodes were mashed in PBS and filtered through a 60µm nylon mesh to obtain single cell suspensions. Tumor and lymph node cells were incubated with Fc block (LICR) for 15-20min at 4°C prior staining with monoclonal antibodies.

Tumor cells were stained for cell surface antigens with the following monoclonal antibodies: CD45-APC (clone: M1/89, LICR), CD11b-pacific blue (M1/70, LICR), CD11c-PerCP-Cy5.5 (N418, eBioscience™), MHC-II-BV785 (M5/114.15.2, BioLegend), Siglec-F-superbright600 (1RNM44N; eBioscience™) F4/80-APC-eFluor780 (BM8, eBioscience™), Ly6C-BV711 (HK1.4, Biolegend), Ly6G-BUV395 (1A8, BD Biosciences), CD80-PE-Cy7 (16-10A1, BioLegend). Cells from lymph nodes were stained for cell surface antigens with the following monoclonal antibodies: CD3-BUV737 (145-2C11, BD Biosciences) CD4-BUV395 (RM4-5, BD Biosciences), CD8-APC-eFluor780 (53-6-7, eBioscience™), CD11b-pacific blue (M1/70, LICR), CD62-L-PerCP-Cy5.5 (MEL-14, eBioscience™), CD44-BV605 (IM7, Biolegend), MHC-II-BV785 (M5/114.15.2, BioLegend), CD11c-APC (N418, eBioscience™), CD279/PD-1-Super Bright645 (J43, eBioscience™)s, CD69-BV711 (H1.2F3, BioLegend), CD80-PE-Cy7 (16-10A1, BioLegend). Intracellular staining was performed with Ki-67-PE-Dazzle594 (16A8, BioLegend) and Foxp3-PE (FJK-16s, eBioscience™) antibodies. Tumor and lymph node cells were subjected to cell surface antigen staining for 30-40 minutes at 4°C. For the exclusion of non-viable cells, LIVE/DEAD™ fixable aqua dead cell stain kit (L34966, Thermo Fisher Scientific) was used. Cells were then fixed and permeabilized with Foxp3 transcription factor staining buffer set (00-5223-56, Thermo Fisher Scientific) as per the manufacturer’s instructions. Lymph node cells were further subjected to Ki-67 and Foxp3 intracellular staining for 30-40 minutes at room temperature. Cells were washed and re-suspended in PBS, and filtered through 60µm nylon mesh for acquisition by flow cytometry. Sample acquisition was performed on BD Fortessa or CytoFLEX LX analyser, and the data was analyzed using FlowJo software (v10.2).

### Elispot

Spleens were isolated from mice at 13-14 days post B16F10 cell transplant or 7-8 days post-cryoablation. Spleens were mashed, subjected to RBC lysis, and single cell suspensions were prepared in a T-cell media comprising RPMI media (61870-010, gibco) with 10% FBS, 1% pencillin-streptomycin, 1% minimal essential media (11140-035, gibco), 1% sodium pyruvate (11360-039, gibco), 1%HEPES (15630-056, gibco) and 0.1% beta-mercapitoethanol (11140-035, gibco). Splenocytes were cultured at 5% CO_2_ and 37°C. At day 3 and 6 of culture, 300units/ml of IL-2 (212-12, peprotech) was added to stimulate the proliferation of T-cells. On day 8, cultured cells were harvested and subjected to CD8^+^ T-cell enrichment by CD8 T-cell isolation kit (130-104-075, Miltenyi Biotec). CD8^+^ T-cells were seeded into 96-well Interferon-γ (IFN-γ) elispot plates at 200,000 cells/well. Murine trp-2 peptide (SVYDFFVWL, LICR) of 0.2µg was added to each well as a tumor antigen peptide. For the control wells, ovarian ID-8 tumor derived peptide (YACENGQTDV, LICR) was added as an irrelevant peptide. Freshly isolated and irradiated splenocytes of 10,000 cells/well were added as a source of antigen presenting cells. Elispot assay was performed as per the manufacturer’s instructions (3321-4HST-10, MABTECH).

### Tetramer and Intracellular Cytokine Staining

For tetramer staining to detect the presence of antigen specific CD8^+^T-cells, single cell suspensions of 1-5million cells from spleen were subjected to Fc blocking, and stained at room temperature with Trp-2-BV421 (TB-5004-4, MBL International) and gp-100-PE (TB-M505-1, MBL International) tetramers for 30min. The cells were then stained with CD19-APC/Cy7 (6D5, Biolegend), CD4-BUV395 and CD8-FITC (KT15, Invitrogen) antibodies, along with LIVE/DEAD™ fixable aqua dye at 4°C for 30min. Cells were then fixed with Foxp3 transcription factor staining buffer set prior flow cytometry analysis.

For the detection of intracellular cytokines, 1-5million cells obtained from spleen were subjected to *in vitro* stimulation with 50 ng/ml of phorbol 12-myristate 13-acetate (P8139, Sigma-Aldrich) and 750 ng/ml ionomycin (407952, Sigma-Aldrich) for 6 hours at 37°C, 5% CO2 in the T-cell media (as described for elispot). 1X Brefeldin-A (420601, BioLegend) was added in the last 4 hours to prevent the secretion of cytokines. After 6 hours, cells were subjected to Fc blocking, followed by staining with CD3-BUV737, CD8-APC-eFluor780, CD4-BUV395 antibodies and LIVE/DEAD™ fixable aqua dye at 4°C for 30min. Cells were then fixed and permeabilized with Foxp3 transcription factor staining buffer set, intracellularly stained with IFN-γ-PE (XMG1.2, eBioscience™) and TNF-α-APC (MP6-XT22, eBioscience™) antibodies. Isotype control staining was done with IgG1 kappa antibody (eBRG1, eBioscience™). Sample processing post staining, and data acquisition by flow cytometry was performed as mentioned above.

### Histology and Immunohistochemistry

Hematoxylin and eosin (H&E) and caspase-3 staining was performed on 5µm sections of formalin-fixed paraffin-embedded tumor tissues. Sections were briefly incubated in xylene and a gradient of alcohol solutions (100%, 95%, 80%) followed by H_2_0 for deparaffinization. They were then stained with hematoxylin for 4 minutes followed by washing with H_2_0 and incubation in 1% acid-alcohol (1% of 37%HCl, and 70% alcohol) for 3 seconds. The sections were washed again and subjected to eosin staining for 30 seconds, washed, dehydrated and mounted for image analysis. For cleaved caspase-3 staining, sections were prior subjected to melanin removal by treatment with potassium permanganate and oxalic acid (13), followed by staining as per the manufacturer’s instructions (#9661, Cell Signaling).

H&E stained slides were used for tumor morphology and quantification of tumor necrosis. Pathologic tumor necrosis (H&E) and apoptosis (cleaved caspase-3 positive cells) were quantified in representative tumor slides by experienced liver pathologists (J.D. & C.S.) who were blinded to all data.

Tumor slides were analyzed by microscopy. The total surface area was quantified and determination of percentage of tumor necrosis was assessed as a percentage of the total area of the tumor present within the histological sections. Tumor necrosis was assessed by increments of 10%, blindly and independently by two pathologists, at different time points. If discordant, cases were reviewed and quantified as a mean value.

### Statistical Analysis

Statistical analyses were performed using GraphPad Prism software (v7.03, La Jolla, CA). Data are represented as mean ± standard deviation. Statistical comparisons of the data sets were performed using the Mann-Whitney U test. Kaplan-Meier survival curves were compared by log-rank (Mantel-Cox) test. A two-tailed *P*-value of < 0.05 was considered statistically significant.

## Results

### Rate of Freeze Influences Locoregional Tumor Growth and Survival

**Figure 1:**
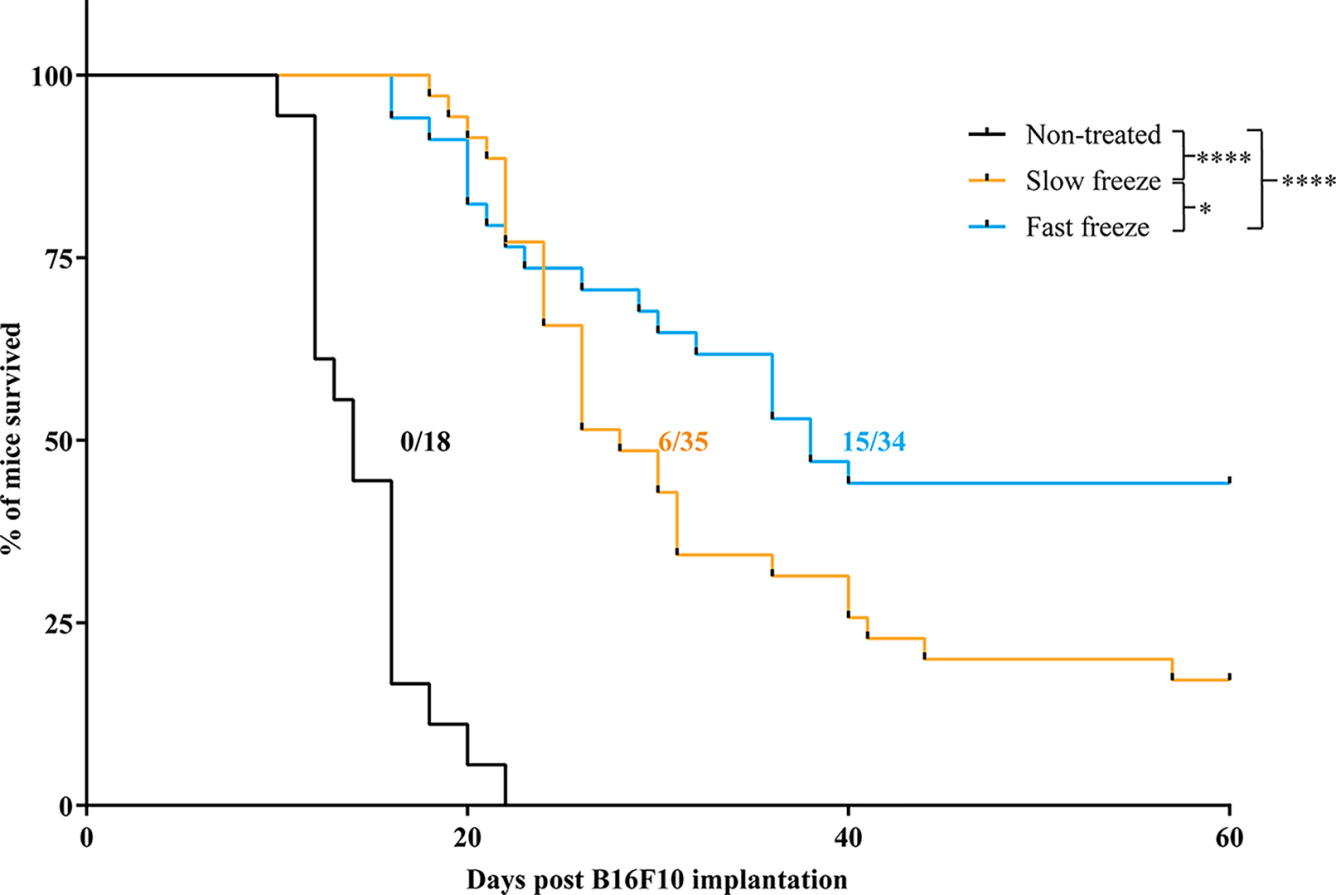
Survival of melanoma bearing mice treated with cryoablation at slow freeze or fast freeze rates. Mice were transplanted with 5 x 10^6^ B16F10 melanoma cells sub-cutaneously on day 1 and received cryoablation between days 7-9. Kaplan-Meier survival curves of mice treated with slow freeze or fast freeze, along with non-treated controls. Data obtained from four independent experiments. Non-treated controls (n = 18), slow freeze (n = 35), fast freeze (n = 34). Numbers displayed on the graph indicate the no. of tumor-free surviving mice/total no. of mice employed for each group. Survival curves were analyzed using log-rank (Mantel-Cox) test. *P < 0.05, ****P < 0.0001.

We evaluated the effectiveness of fast and slow freeze rates in their ability to locally ablate the tumor in a B16F10 model of melanoma. Cryoablation by either protocols resulted in a significant delay of tumor growth and enhancement of survival over non-treated controls (**Figure 1** and **Supplementary Figure 1**). Furthermore, mice treated with fast freeze protocol resulted in an improved survival over slow freeze protocol; 44% of mice were alive and tumor-free at the end of the 2month follow-up period when treated with the fast freeze protocol *vs* 17% when treated with the slow freeze protocol (*P*-value <0.05; **Figure 1**). Overall, these results demonstrate that fast freeze outperforms slow freeze in its ability to locoregionally treat melanoma tumors.

### Rate of Freeze Influences Tumor Necrosis but Does Not Induce Apoptosis

**Figure 2:**
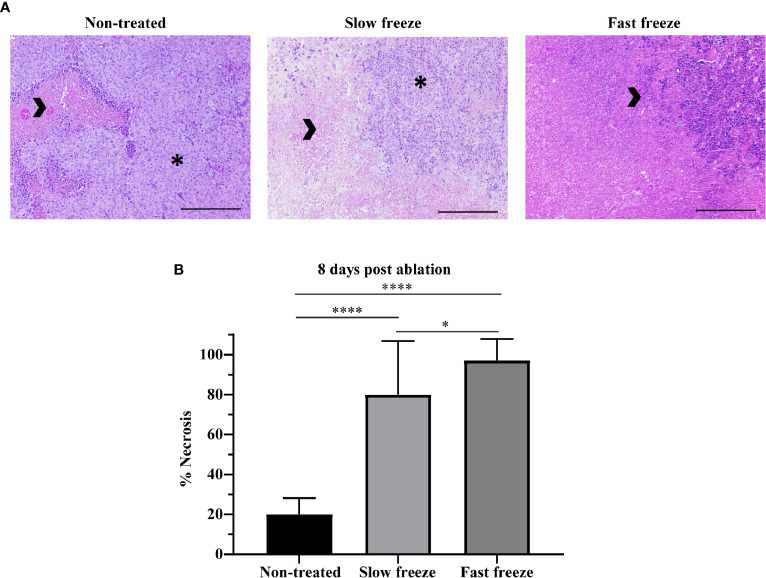
Impact of differential freeze rate on tumor necrosis. **(A)** Representative images of H&E staining of tumor tissue sections obtained at 8 days post cryoablation, along with non-treated tumor controls. Images acquired at 10X magnification. Arrowheads indicate necrotic tissue, and asterisks indicate viable tumor. Scale represents 1000µm. **(B)** Quantification of tumor necrosis in non-treated and cryoablated tumor tissues. Data plotted as mean and ± S.D. obtained from two independent experiments (n = 7-14). Data was analyzed using non-parametric Mann-Whitney U test. *P < 0.05, ****P < 0.0001.

Survival outcomes obtained from differential freeze rates prompted us to determine the extent of necrosis induced in the tumors, examined at 8 days post-cryoablation by H&E staining. Apart from the spontaneous necrosis observed in the non-treated mice, we observed a significantly increased ablation-induced necrosis, with markedly higher necrosis in fast freeze as compared to slow freeze groups (**Figure 2**), corroborating the data obtained from survival experiments. Treatment-induced apoptosis, as measured by cleaved caspase-3 staining, was not observed at the periphery of the ablated zone (**Supplementary Figure 2**). Interestingly, cleaved caspase-3 positive cells were present at the limiting plate zone between spontaneous tumor necrosis and viable tissue in particular in the non-treated tumors (**Supplementary Figure 2**). Taken together, these results show that apoptosis may be observed at the rim of spontaneous necrosis in melanoma tumors, and that cryoablation besides causing tumor necrosis, did not induce apoptosis at the periphery of the ablated zone.

### Cryoablation Modulates the Immune Landscape in TDLN and TNDLN

**Figure 3:**
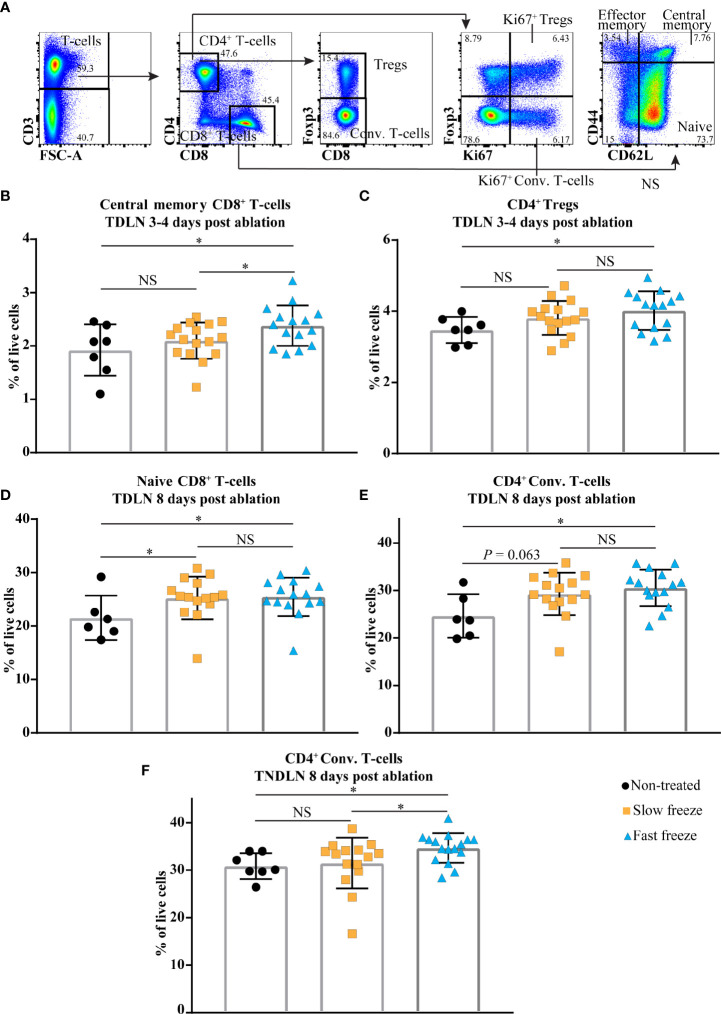
Cryoablation-induced changes in the immune cell compartments in local tumor draining lymph node (TDLN) and distant contralateral tumor non-draining lymph node (TNDLN). **(A)** Flow cytometry gating strategies employed to characterize CD4^+^ and CD8^+^ T-cell subsets. **(B, C)** Percentages of CD8^+^ central memory T-cells and CD4^+^ regulatory T-cells (Tregs) within live cells in TDLN at day 3-4 post cryoablation. **(D, E)** Percentages of CD8^+^ naive T-cells and CD4^+^ conventional (Conv.) T-cells within live cells in TDLN at day 8 post cryoablation. **(F)** Percentages of CD4^+^ Conv. T-cells within live cells in TNDLN at day 8 post cryoablation. Data plotted as mean and ± S.D. obtained from four independent experiments (n = 6-16). Each data point represents an individual mouse. Data was analyzed using non-parametric Mann-Whitney U test. *P < 0.05, NS, not significant.

We next tested the ability of slow and fast freeze protocols to initiate local and systemic immune responses, by performing extensive flow cytometry analyses of various immune cell subsets in lymph nodes and tumors (**Supplementary Tables 1**–**6**). T-cell populations in the lymph nodes were characterized by flow cytometry based on the expression of CD4, CD8, CD62L, CD44, Foxp3 and Ki67 markers (**Figure 3A**). In the local tumor draining lymph node (TDLN), at day 3-4 post cryoablation, increased frequencies of CD8^+^ central memory T-cells and Foxp3^+^ CD4^+^ regulatory T-cells (Tregs) were observed only in the fast freeze group as compared to non-treated controls. In addition, central memory subset of CD8^+^ T-cells were also significantly higher in fast freeze group as compared to slow freeze group (**Figures 3B, C**). The increased frequencies in these T-cell subsets was transient, as it was not observed at a later time point (i.e. day 8) (**Supplementary Tables 1** and **2**). At day 8 post cryoablation, naive CD8^+^ T-cell frequencies in the TDLN were significantly increased in both slow and fast freeze groups (**Figure 3D**). Interestingly, at 8 days post cryoablation, frequencies of CD4^+^ conventional T-cells and their proliferation status were significantly increased in both the TDLN and the contralateral tumor non-draining lymph node (TNDLN) only in the fast freeze group (**Figures 3E, F** and **Supplementary Tables 2**, **4**), indicative of systemic immune responses. Of note, a clear trend was observed with decreased PD1^+^ CD4^+^ Tregs at day 8 post ablation in TDLN in both slow and fast freeze groups (*P*=0.076 and *P*=0.082, respectively **Supplementary Table 2**).

Interestingly, we also observed increased frequencies of migratory and conventional (resident) dendritic cell subsets in both fast and slow freeze groups *vs* non-treated controls, in the TNDLN at day 8 post treatment (**Supplementary Table 4**). No difference in DCs frequencies was found between both cryoablation groups and controls in TDLN. This could be due to the fact that the tumor bearing control mice had a high percentage of DCs in the local TDLN due to the presence of a growing tumor and its associated inflammation. As a result, we did not observe a further increase in DCs in TDLN post cryoablation when compared to controls.

Collectively, these results indicate that cryoablation (irrespective of the protocol) was able to initiate local immune responses as shown by increased frequencies of CD8^+^ T-cells in TDLN at day 8 post treatment. However, the conventional CD4^+^ conventional T-cells (non-Tregs) were increased only in the fast freeze group, both in the local TDLN and distant TNDLN, suggestive of systemic immune responses. This indicates that fast freeze could be advantageous over slow freeze in eliciting immune responses.

### Myeloid Cells Infiltrate the Tumors Early After Cryoablation

**Figure 4:**
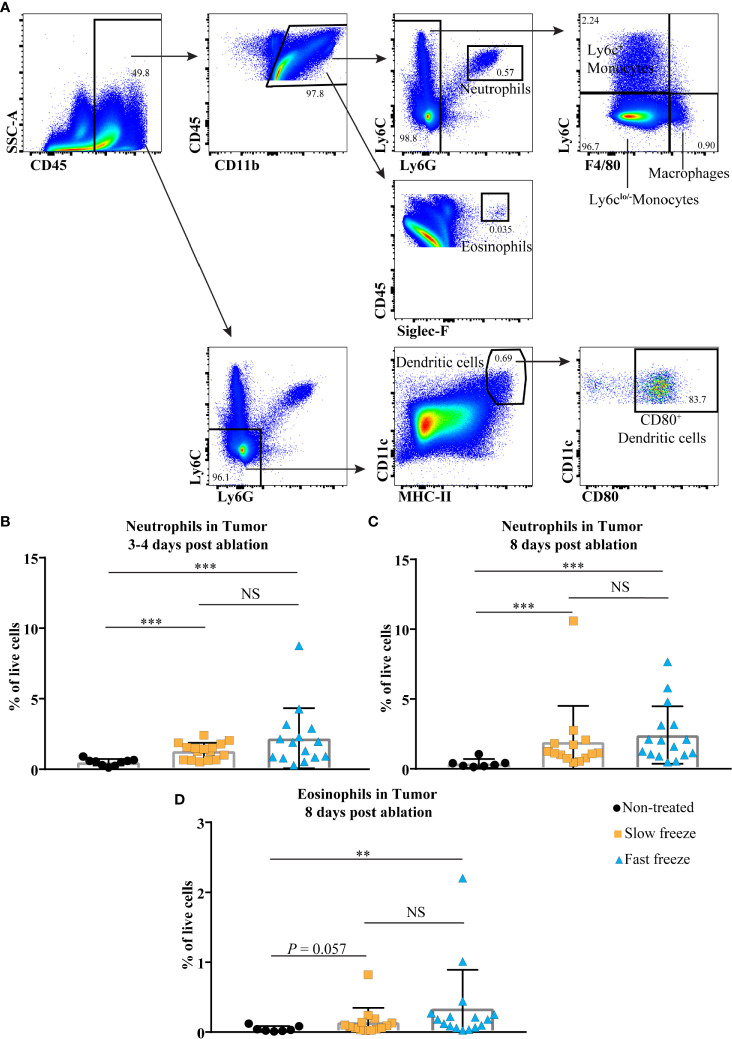
Cryoablation-induced changes in the tumor infiltrating myeloid cells. **(A)** Flow cytometry gating strategies employed to characterize tumor infiltrating myeloid cells. **(B, C)** Percentages of neutrophils within live cells at days 3-4 and 8 post cryoablation. **(D)** Percentages of eosinophils within live cells at day 8 post cryoablation. Data plotted as mean and ± S.D. obtained from four independent experiments (n = 7-16). Each data point represents an individual mouse. Data was analyzed using non-parametric Mann-Whitney U test. **P < 0.01, ***P < 0.001. NS, not significant.

We also examined for changes in the myeloid cell composition in tumors post cryoablation (**Figure 4A**). Neutrophils, known as the primary responders of infection and injury (14), were increased at day 3-4 and 8 post cryoablation in both treatment groups (**Figures 4B, C**). Intriguingly, at day 8 post ablation, eosinophils were significantly increased in the fast freeze group, and exhibited an increasing trend in the slow freeze group (**Figure 4D**). Frequencies of other cell types such as DCs either decreased (in particular in the first days following ablation, reflecting treatment-related cell death) or remained unchanged such as monocytes and macrophages (**Supplementary Tables 5** and **6**).

### Cryoablation With Fast Freeze Rate Improves Survival Post Re-Challenge

**Figure 5:**
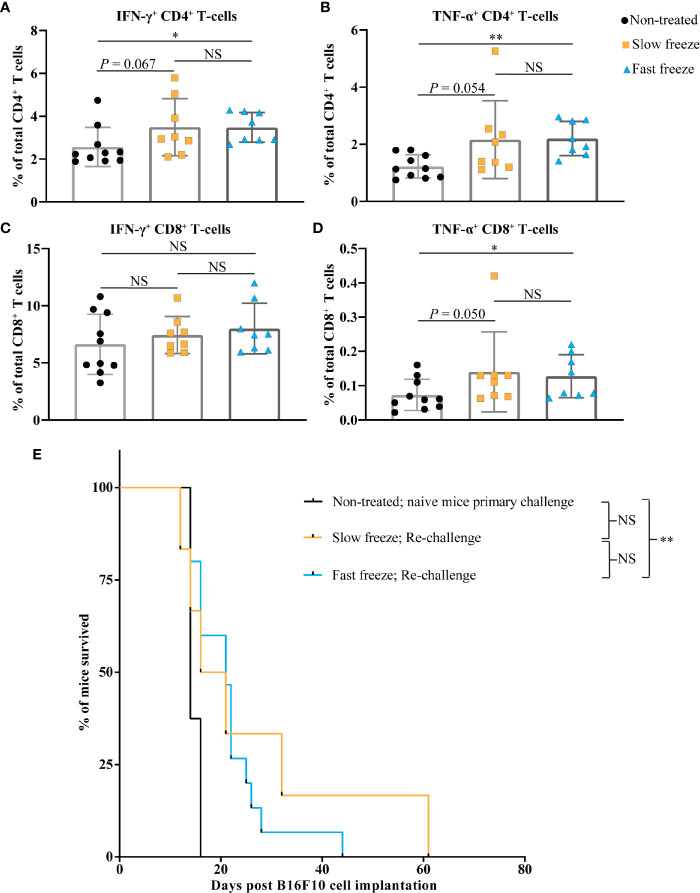
Cryoablation by fast freeze rate induces immune responses and extends the survival post re-challenge. **(A–D)** Spleens isolated at day 7-8 post cryoablation, along with non-treated controls. Splenocytes stimulated *in vitro* for 6 hours with PMA and ionomycin. **(A, B)** Percentages of interferon-γ^+^ and tumor necrosis factor-α^+^ cells within total CD4^+^ T-cell fraction. **(C, D)** Percentages of interferon-γ^+^ and tumor necrosis factor-α^+^ cells within total CD8^+^ T-cell fraction. Data plotted as mean and ± S.D. obtained from three independent experiments (n=8-10). **(E)** Cryoblation-treated tumor-free surviving mice were subjected to re-challenge with 5 x 10^6^ B16F10 cells on the contralateral flank between 60-70 days post primary melanoma cell transplant. Kaplan-Meier survival curves of mice post re-challenge, which previously received fast freeze (n=15) or slow freeze (n=6) ablation treatments, along with naive mice (n=8) that received melanoma cells for the first time. Data plotted was obtained from three independent experiments, and analyzed using log-rank (Mantel-Cox) test. *P < 0.05, **P < 0.01, NS, not significant.

In order to investigate the systemic immunological impact of cryoablation, splenocytes at 8 days post ablation were stimulated *in vitro* with PMA and ionomycin. Frequencies of IFN-γ expressing CD4^+^, and TNF-α expressing CD4^+^ and CD8^+^ T-cells were significantly higher in fast freeze group as compared to non-treated controls. An increasing trend was also observed in the slow freeze group in comparison to non-treated controls. No differences were observed between the groups in the frequency of IFN-γ expressing CD8^+^ T-cells (**Figures 5A–D**). Tetramer staining of splenocytes for the detection of Gp-100 and Trp-2 antigen specific CD8^+^ T-cells did not reveal any significant differences between the non-treated and the cryoablated groups. Similar results were also obtained from IFN-γ elispot assay of splenic CD8^+^ T-cells with Trp-2 antigen (**Supplementary Figure 3**). In addition, we also did not observe any significant increase in the systemic levels of investigated plasma cytokines at day 3 or day 8 post cryoablation in comparison to non-treated controls (data not shown).

As a final step, mice that were treated with cryoablation and remained tumor-free for at least 2 months from the time of initial B16F10 transplant were re-challenged with the same cells on their contralateral flank. Significantly more mice treated with the fast freeze reached this point than the ones treated with the slow freeze protocol (15 *vs* 6 mice), and were subsequently used for re-challenge experiments. In comparison to the naïve mice that received melanoma cells for the first time, re-challenged mice only from the fast freeze group survived significantly longer [median overall survival: 14 *vs* 21 days, respectively (*P* = 0.003); **Figure 5E** and **Supplementary Figure 4**]. This suggests the generation of immunological memory responses against tumors, that restricted the tumor growth and extended the survival post re-challenge.

## Discussion

The main finding of this study is that cryoablation with fast freeze protocol achieved strong anticancer effects locoregionally in the treated tumor with enhanced survival in a mouse model of melanoma. Moreover, our data obtained from a combination of *ex vivo* immune cell characterization and *in vivo* re-challenge experiments demonstrates favorable systemic immunogenic properties of the fast freeze rate.

Rate of freeze is a critical parameter of the local effectiveness of cryoablation. Fast freeze protocols achieve higher levels of intracellular ice formation with greater causation of tissue damage (1). This lead to their wide implementation in the clinic where successful *in situ* eradication of small cancer lesions was observed. However, when cryoablation is to be used as an *in vivo* vaccination tool, the variation in the mechanistic processes of fast and slow freeze rates on cell injury may influence the balance of necrosis/apoptosis, and the quality of the immune responses (11, 12, 15). There are reports indicating that tumor necrosis elicits immunogenic responses (16, 17), whereas some contradicting studies suggest that apoptosis (as opposed to necrosis) is a strong driver of anti-tumor immune responses (18, 19). A previous study by Sabel et al. reported the advantage of fast freeze over slow freeze, but information regarding necrosis/apoptosis in the ablated tissue, and an in-depth characterization of immune responses appear lacking (11). Our study focused on addressing these aspects with an aim of choosing the right protocol that would augment the impact of targeted immunotherapies employed in combination with cryoablation in clinical settings.

We first tested the impact of fast and slow freeze in their ability to eradicate the tumor locally. Our results demonstrated that fast freeze protocol ablated the tumor effectively and improved the survival as compared to slow freeze, confirming previously observed results in a breast cancer model (11). As a next step, we determined the extent of apoptosis and necrosis induced in the tumor by slow and fast freeze protocols. On day 8 post ablation, we did not observe any treatment-induced apoptosis, even at the periphery of the ablation zone, which has been suggested to undergo apoptosis (15). Our data suggests that the cryoablated tumor tissue predominantly dies by necrosis, with rapid freeze rate inducing a greater extent of tissue necrosis than slow freeze rate. The presence of remaining viable tumor tissue in the slow freeze group is likely to have resulted in tumor re-growth post ablation, and therefore, a significantly reduced survival as compared to the fast freeze group.

In order to see if the difference in the extent of necrosis observed between fast and slow freeze is associated with a variation in the degree of immune responses, we studied the immune cell composition in local TDLN, distant contralateral TNDLN, and tumors, post ablation. At day 3-4 post treatment, fast freeze mode of ablation resulted in a transient increase of CD8^+^ central memory T-cell proportions in TDLN, in comparison to slow freeze and non-treated control groups. In fact, this brief time window seems not enough for the generation of memory responses. It can be explained by the presence of an already existing memory T-cell repertoire specific to tumor-associated melanoma antigens. In line with our data, a recent study on healthy human blood samples revealed the presence of a low affinity CD8^+^ T-cell pool specific to melanoma antigens, referred to as natural autoreactive T-cells. The behavior exhibited by these T-cells upon melanoma antigen stimulation *ex-vivo* implied that they belong to the central memory pool of T-cells (20). Cryoablation with fast freeze could have led to the increased frequency of these pre-existing memory cells, due to the increased availability of tumor antigens. We have also observed a concomitant increase in Tregs in the fast freeze group, which could indicate an immunoregulatory mechanism in place to counteract the autoreactive CD8^+^ T-cells. These results may also encourage the application of anti-CTLA4 therapy that could selectively deplete the Tregs (21), and upon co-application with cryoablation is likely to result in an improved anti-tumor immunity, as demonstrated previously (22, 23). Both the modes of cryoablation resulted in increased frequencies of naïve CD8^+^ T-cells in TDLNs at day 8 post treatment. This was also accompanied in the fast freeze group by an increased frequency of CD4^+^ T-cells and their proliferative status, both in TDLN and TNDLN, indicative of local and systemic immune responses. In the distant contralateral TNDLN, the percentage of DC subsets was increased in both cryoablation groups as compared to controls. These results suggest that fast freeze evokes relatively stronger immune responses in comparison to slow freeze, both at local and systemic levels.

Increased proportion of neutrophils was observed in the tumor immune infiltrate at both time points post cryoablation, as neutrophils are known to be the initial entrants to the site of injury. Data from a recent study suggests that neutrophils are rapidly mobilized from the bone marrow to the ablated site, as demonstrated by a drop of neutrophils in the bone marrow and a corresponding spike in the peripheral blood by 24hrs post ablation (24). In addition, we also observed an increased frequency of eosinophils in the tumors at day 8 post ablation. Tumor infiltrating eosinophils are known to secrete cytokines and chemokines to attract T-cells for combating the tumors (25). Indeed, eosinophil infiltration into tumors has been shown to positively correlate with patient survival in many cancers including melanoma (26). Future studies should investigate if cryoablation of a tumor triggers eosinophil infiltration in distant, non-treated tumor sites.

Increased frequencies of TNF-α and IFN-γ producing CD4^+^ T-cells and TNF-α producing CD8^+^ T-cells were observed in the fast freeze group when compared to non-treated controls, when the splenocytes were *in vitro* stimulated with PMA and ionomycin. An increasing trend was also observed in the slow freeze group. These results suggest that the splenic T-cells from the cryoablated mice were immunologically activated due to ablation, and therefore, had a higher percentage of TNF-α and IFN-γ producing T-cells upon *in vitro* stimulation. This data further underscores the induction of systemic immunity as a result of cryoablation, in particular, with the fast freeze protocol. Intriguingly, we neither observed a systemic increase of Gp-100 or Trp-2 antigen specific CD8^+^ T-cells nor an increased production of IFN-γ by splenic CD8^+^ T-cells stimulated with a Trp-2 antigen *in vitro*. This could indicate that Gp-100 and Trp-2 tumor-associated antigens might not have an important role to play in the induction of systemic immunity post cryoablation. An alternative explanation could be that the frequencies of Gp-100 and Trp-2 antigen specific T-cells are too low to be detected in our experimental system. We also did not observe any significant increase in the systemic levels of plasma cytokines post cryoablation.

As a last step, we re-challenged the tumor-free surviving mice post cryoablation with a high dose of 5 x 10^6^ B16F10 melanoma cells in the opposite flank. As compared to naive mice, significantly improved survival was only observed in the group that was prior treated with the fast freeze protocol, but not with the slow freeze protocol. This also corroborates the differences in the observed immune responses between the fast and slow freeze treatments. In a similar previous study with B16OVA tumors, re-challenge of tumor-free mice that were prior treated with cryoablation resulted in 40-50% long-term survivors that completely rejected the tumor (22). In our study, all the re-challenged mice of the fast freeze group died at a later time point, without any long-term survivors. The difference in the outcomes could be due to the fact that the previous study employed B16OVA cell line, which is more immunogenic than the B16F10 cell line employed in our study. More importantly, re-challenge was performed with 15 x 10^3^ cells in the previous study as compared to 5 x 10^6^ cells used in our study, indicating that the ability to completely reject the tumor cells in a re-challenge experiment also varies with the number of cells implanted.

In conclusion, cryoablation with fast freeze rate should be preferred over slow freeze rate owing to the locoregional tumor control and the associated immune responses. Therefore, cryoablation with a fast freeze rate should be applied in future for the combinatorial application of cryoablation with targeted immunotherapies.

## Data Availability Statement

The original contributions presented in the study are included in the article/**Supplementary Material**. Further inquiries can be directed to the corresponding author.

## Ethics Statement

The animal study was reviewed and approved by Service de la Consommation et des Affaires Vétérinaires, Vaud, Switzerland.

## Author Contributions

CY and RD designed the study, performed the experiments, analyzed the data and wrote the manuscript. JD, CS, CC, BK, AD, and LK analyzed the data. All authors contributed to the article and approved the submitted version.

## Funding

This study received funding from BTG/Boston Scientific. The funder was not involved in the study design, collection, analysis, interpretation of data, the writing of this article or the decision to submit it for publication.

## Conflict of Interest

RD: Consultant: Guerbet, Boston Scientific/BTG. Grant Support: Guerbet, Boston Scientific/BTG, Society of Interventional Oncology. BK is an employee of Boston Scientific/BTG.

The remaining authors declare that the research was conducted in the absence of any commercial or financial relationships that could be construed as a potential conflict of interest.

## Supplementary Material

Supplementary Figure 1 Tumor growth kinetics of melanoma bearing mice treated with cryoablation at slow freeze or fast freeze rates. Mice were transplanted with 5 x 10^6^ B16F10 melanoma cells sub-cutaneously on day 1 and received cryoablation between days 7-9. Tumor growth curves of each individual mouse from **(A)** non-treated (n=18), **(B)** slow freeze (n=35), and **(C)** fast freeze (n=34) groups. Data pooled from four independent experiments.

Supplementary Figure 2 Cryoablation does not induce apoptosis. Cleaved caspase-3 staining of tumor tissue sections obtained from **(A)** non-treated controls, and 8 days post **(B)** slow freeze or **(C)** fast freeze treatment protocols. Images acquired at 10X magnification. Arrowheads indicate cleaved-caspase-3 positive cells, and asterisks indicate the limiting plate between viable and necrotic tissue. Scale represents 1000µm. Representative data obtained from two independent experiments (n=7-14).

Supplementary Figure 3 Immune responses against Gp-100 and Trp-2 antigens post cryoablation. Spleens isolated from non-treated controls and at day 7-8 post cryoablation. Percentages of **(A)** Gp-100 specific and **(B)** Trp-2 specific T-cells within the total CD8^+^ T-cell fraction. **(C)** Elispot-based quantification of interferon-γ production by splenic CD8^+^ T-cells incubated *in vitro* with murine Trp-2 (SVYDFFVWL) antigen. Representative data obtained from three independent experiments (n=8-11). NS, not significant.

Supplementary Figure 4 Tumor growth kinetics post re-challenge. Cryoblation-treated, tumor-free, long-term surviving mice were subjected to re-challenge with 5 x 10^6^ B16F10 cells on the contralateral flank between 60-70 days post primary B16F10 cell transplant. Tumor growth curves of each individual mouse from **(A)** Naive control mice that received melanoma cells for the first time (n=8); re-challenged mice from **(B)** slow freeze (n=6), and **(C)** fast freeze (n=15) groups. Data pooled from three independent experiments.

Supplementary Table 1 Differences in percentages of immune cell populations between experimental groups at day 3-4 post cryoablation in tumor draining lymph node (TDLN). Data was analyzed using non-parametric Mann-Whitney U test. NS, not significant. Higher (↑) or lower (↓) post cryoablation than non-treated controls (columns 1 and 2). Higher (↑) or lower (↓) post fast freeze than slow freeze group (column 3).

Supplementary Table 2 Differences in percentages of immune cell populations between experimental groups at day 8 post cryoablation in tumor draining lymph node (TDLN). Data was analyzed using non-parametric Mann-Whitney U test. NS, not significant. Higher (↑) or lower (↓) post cryoablation than non-treated controls (columns 1 and 2). Higher (↑) or lower (↓) post fast freeze than slow freeze group (column 3).

Supplementary Table 3 Differences in percentages of immune cell populations between experimental groups at day 3-4 post cryoablation in tumor non-draining lymph node (TNDLN). Data was analyzed using non-parametric Mann-Whitney U test. NS, not significant. Higher (↑) or lower (↓) post ablation than non-treated controls (columns 1 and 2). Higher (↑) or lower (↓) post fast freeze than slow freeze group (column 3).

Supplementary Table 4 Differences in percentages of immune cell populations between experimental groups at day 8 post cryoablation in tumor non-draining lymph node (TNDLN). Data was analyzed using non-parametric Mann-Whitney U test. NS, not significant. Higher (↑) or lower (↓) post cryoablation than non-treated controls (columns 1 and 2). Higher (↑) or lower (↓) post fast freeze than slow freeze group (column 3).

Supplementary Table 5 Differences in percentages of immune cell populations between experimental groups at day 3-4 post cryoablation in tumors. Data was analyzed using non-parametric Mann-Whitney U test. NS, not significant. Higher (↑) or lower (↓) post cryoablation than non-treated controls (columns 1 and 2). Higher (↑) or lower (↓) post fast freeze than slow freeze group (column 3).

Supplementary Table 6 Differences in percentages of immune cell populations between experimental groups at day 8 post cryoablation in tumors. Data was analyzed using non-parametric Mann-Whitney U test. NS, not significant. Higher (↑) or lower (↓) post cryoablation than non-treated controls (columns 1 and 2). Higher (↑) or lower (↓) post fast freeze than slow freeze group (column 3).
