# Supplementary figures and images for: Rate of Freeze Impacts the Survival and Immune Responses Post Cryoablation of Melanoma
Source: Front Immunol. 2021 Jun 3;12:695150. doi: 10.3389/fimmu.2021.695150 (PMC8210778; doi:10.3389/fimmu.2021.695150)

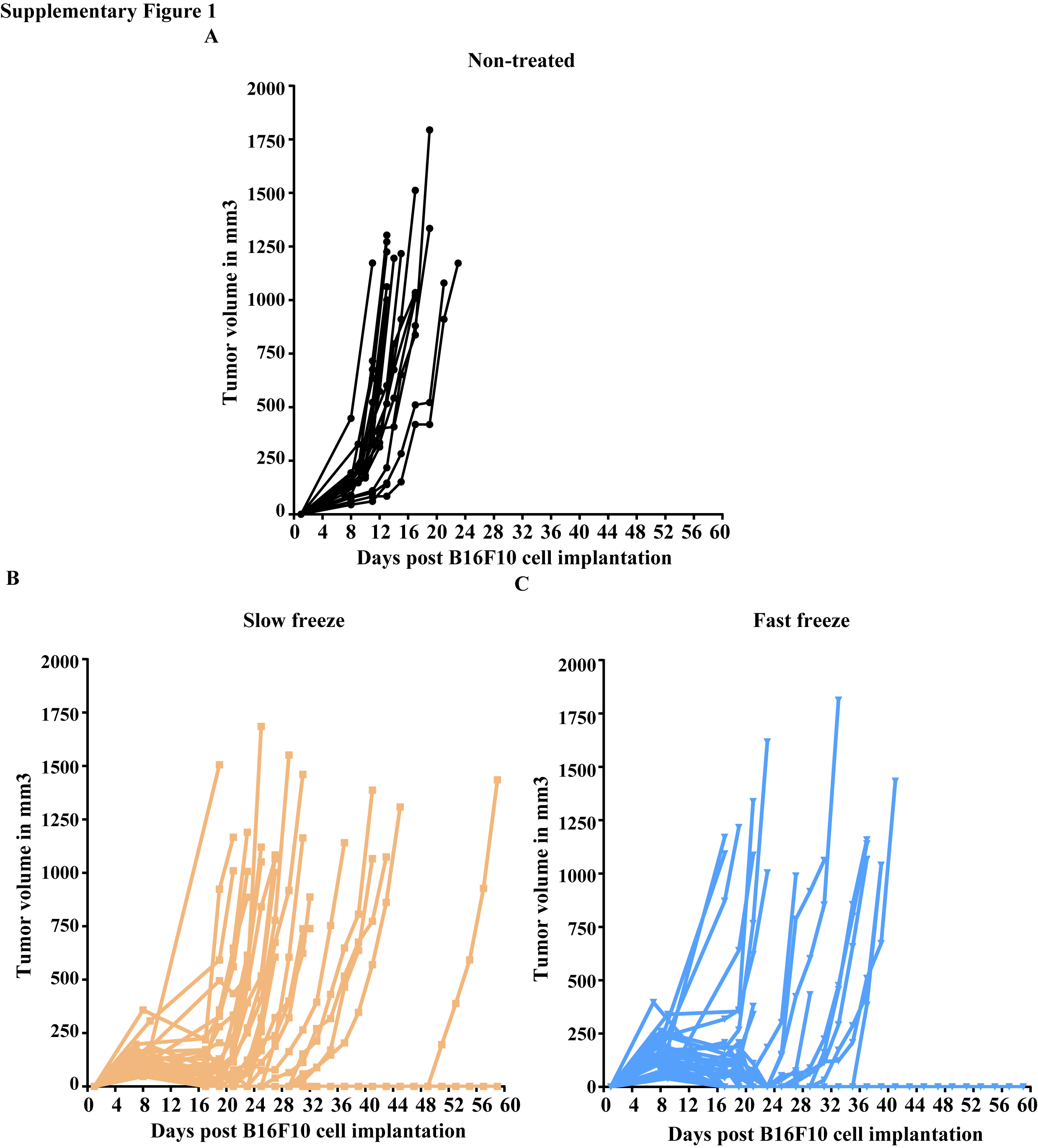

Supplement: Supplementary Figure 1 — Tumor growth kinetics of melanoma bearing mice treated with cryoablation at slow freeze or fast freeze rates. Mice were transplanted with 5 x 10^6^ B16F10 melanoma cells sub-cutaneously on day 1 and received cryoablation between days 7-9. Tumor growth curves of each individual mouse from (A) non-treated (n=18), (B) slow freeze (n=35), and (C) fast freeze (n=34) groups. Data pooled from four independent experiments. [file Image_1.tif]

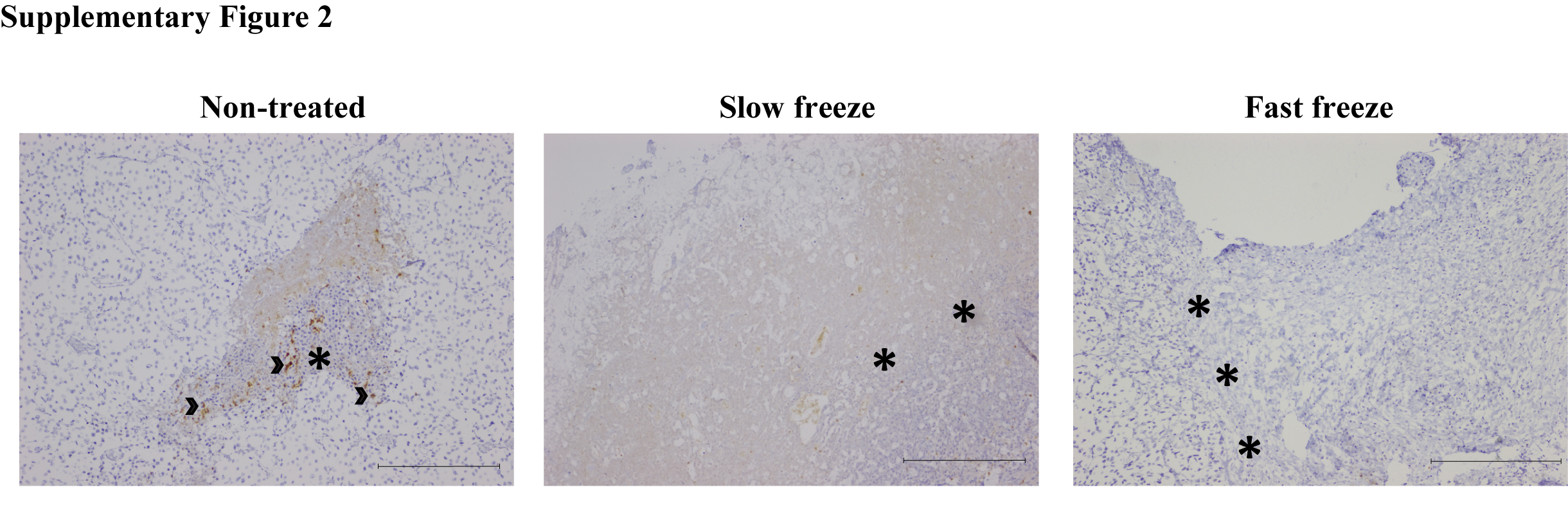

Supplement: Supplementary Figure 2 — Cryoablation does not induce apoptosis. Cleaved caspase-3 staining of tumor tissue sections obtained from (A) non-treated controls, and 8 days post (B) slow freeze or (C) fast freeze treatment protocols. Images acquired at 10X magnification. Arrowheads indicate cleaved-caspase-3 positive cells, and asterisks indicate the limiting plate between viable and necrotic tissue. Scale represents 1000µm. Representative data obtained from two independent experiments (n=7-14). [file Image_2.tif]

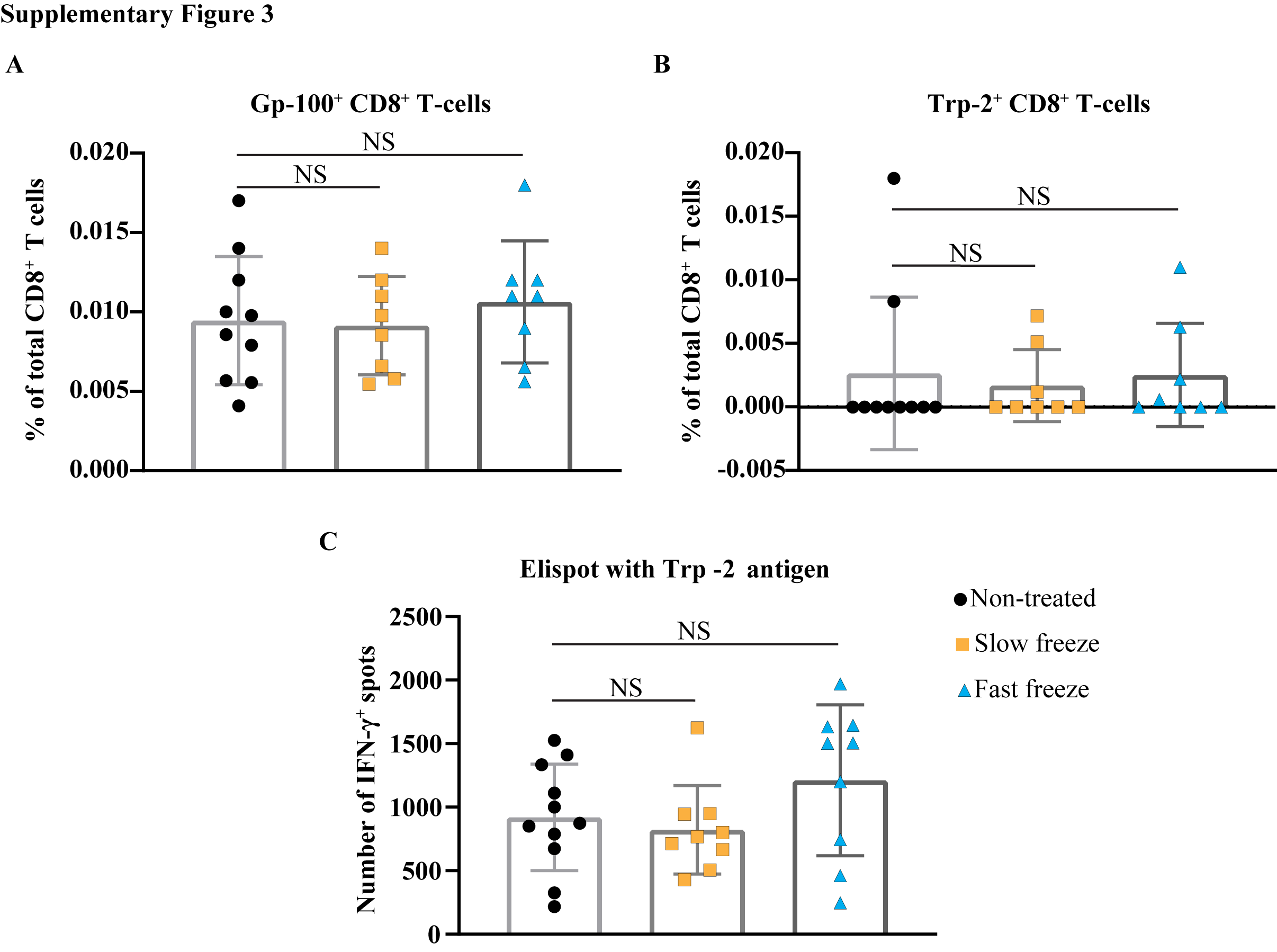

Supplement: Supplementary Figure 3 — Immune responses against Gp-100 and Trp-2 antigens post cryoablation. Spleens isolated from non-treated controls and at day 7-8 post cryoablation. Percentages of (A) Gp-100 specific and (B) Trp-2 specific T-cells within the total CD8+ T-cell fraction. (C) Elispot-based quantification of interferon-γ production by splenic CD8+ T-cells incubated in vitro with murine Trp-2 (SVYDFFVWL) antigen. Representative data obtained from three independent experiments (n=8-11). NS, not significant. [file Image_3.tif]

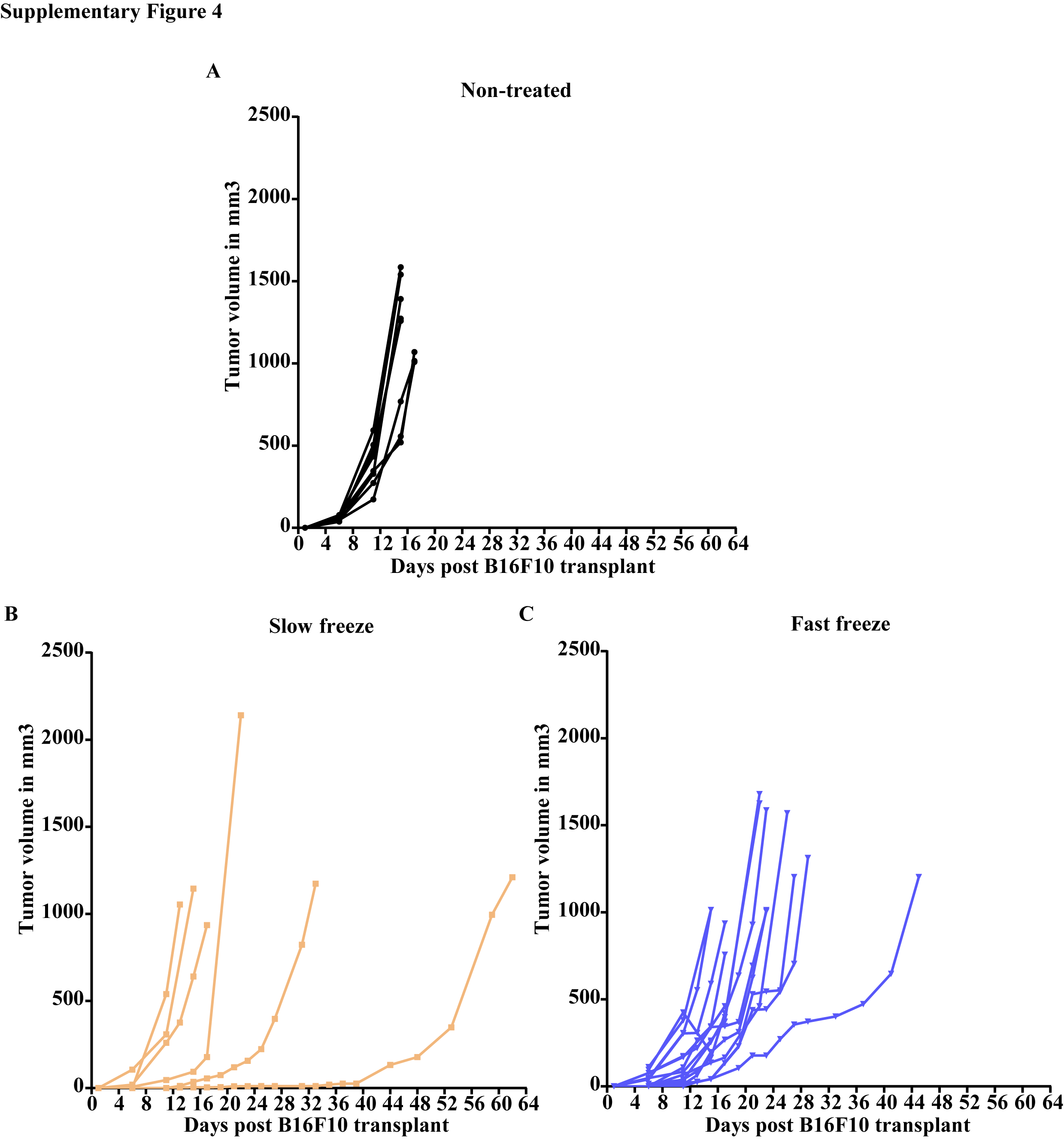

Supplement: Supplementary Figure 4 — Tumor growth kinetics post re-challenge. Cryoblation-treated, tumor-free, long-term surviving mice were subjected to re-challenge with 5 x 10^6^ B16F10 cells on the contralateral flank between 60-70 days post primary B16F10 cell transplant. Tumor growth curves of each individual mouse from (A) Naive control mice that received melanoma cells for the first time (n=8); re-challenged mice from (B) slow freeze (n=6), and (C) fast freeze (n=15) groups. Data pooled from three independent experiments. [file Image_4.tif]
