# Supplementary material for: Rate of Freeze Impacts the Survival and Immune Responses Post Cryoablation of Melanoma
Source: Front Immunol. 2021 Jun 3;12:695150. doi: 10.3389/fimmu.2021.695150 (PMC8210778; doi:10.3389/fimmu.2021.695150)
Supplement: Supplementary Table 4 — Differences in percentages of immune cell populations between experimental groups at day 8 post cryoablation in tumor non-draining lymph node (TNDLN). Data was analyzed using non-parametric Mann-Whitney U test. NS, not significant. Higher (↑) or lower (↓) post cryoablation than non-treated controls (columns 1 and 2). Higher (↑) or lower (↓) post fast freeze than slow freeze group (column 3). [file Table_4.docx]

| **Day 8 post cryoablation TNDLN** | **Slow freeze**  **Vs**  **Non-treated** | **Fast freeze**  **Vs**  **Non-treated** | **Fast freeze  Vs  Slow freeze** |
| --- | --- | --- | --- |
| **Conventional Dendritic cells (cDC)** | ***P*<0.05 ↑** | NS | NS |
| CD80+ cDC | ***P*<0.05 ↑** | ***P*<0.05 ↑** | NS |
| CD11b+ cDC | ***P*<0.01 ↑** | ***P*=0.057 ↑** | NS |
| CD11b- cDC | NS | NS | NS |
| **Migratory Dendritic cells (mDC)** | ***P*<0.05 ↑** | ***P*<0.05 ↑** | NS |
| CD80+ mDC | NS | ***P*<0.05 ↑** | NS |
| CD11b+ mDC | ***P*=0.063 ↑** | ***P*<0.05 ↑** | NS |
| CD11b- mDC | NS | NS | NS |
| **CD4+ Conventional T-cells (Conv. T-cells)** | NS | ***P*<0.05 ↑** | ***P*<0.05 ↑** |
| Ki67+ CD4+ Conv. T-cells | NS | ***P*<0.01 ↑** | NS |
| CD69+ CD4+ Conv. T-cells | NS | NS | NS |
| PD1+ CD4+ Conv. T-cells | NS | NS | NS |
| **CD4+ Regulatory T-cells (Tregs)** | NS | NS | NS |
| Ki67+ CD4+ Tregs | NS | NS | NS |
| CD69+ CD4+ Tregs | NS | NS | NS |
| PD1+ CD4+ Tregs | NS | NS | NS |
| **CD8+ T-cells** | NS | NS | NS |
| Ki67+ CD8+ T-cells | NS | NS | NS |
| CD69+ CD8+ T-cells | NS | NS | NS |
| PD1+ CD8+ T-cells | NS | NS | NS |
| CD62L+ CD44- CD8+ T-cells (Naive) | NS | NS | NS |
| CD62L+ CD44+ CD8+ T-cells (Central memory) | NS | NS | NS |
| CD62L- CD44+ CD8+ T-cells (Effector memory) | NS | NS | NS |
