# Supplementary material for: Rate of Freeze Impacts the Survival and Immune Responses Post Cryoablation of Melanoma
Source: Front Immunol. 2021 Jun 3;12:695150. doi: 10.3389/fimmu.2021.695150 (PMC8210778; doi:10.3389/fimmu.2021.695150)
Supplement: Supplementary Table 6 — Differences in percentages of immune cell populations between experimental groups at day 8 post cryoablation in tumors. Data was analyzed using non-parametric Mann-Whitney U test. NS, not significant. Higher (↑) or lower (↓) post cryoablation than non-treated controls (columns 1 and 2). Higher (↑) or lower (↓) post fast freeze than slow freeze group (column 3). [file Table_6.docx]

| **Day 8 post cryoablation Tumor infiltrate** | **Slow freeze**  **Vs**  **Non-treated** | **Fast freeze**  **Vs**  **Non-treated** | **Fast freeze  Vs  Slow freeze** |
| --- | --- | --- | --- |
| **CD45+** | ***P*<0.05 ↑** | NS | NS |
| **Neutrophils** | ***P*<0.001↑** | ***P*<0.001↑** | NS |
| **Dendritic cells (DC)** | NS | NS | NS |
| CD80+ DC | NS | NS | NS |
| CD11b+ DC | NS | NS | NS |
| CD11b- DC | NS | ***P*<0.05 ↓** | ***P*<0.05 ↓** |
| **Ly6C+ monocytes** | NS | NS | ***P*=0.059 ↓** |
| **Ly6Clo/- monocytes** | NS | NS | NS |
| **Macrophages** | NS | NS | NS |
| **Eosinophils** | ***P*=0.057 ↑** | ***P*<0.01 ↑** | NS |
